# Molten salt synthesis of CrMnFeNi alloy nanopowder passivated by TiO_*x*_–ZrO_*y*_ shell used as a superior catalyst support in liquid-phase hydrogenation†

**DOI:** 10.1039/d3ra01797d

**Published:** 2023-04-05

**Authors:** Yasukazu Kobayashi, Shota Yokoyama, Ryo Shoji

**Affiliations:** a Renewable Energy Research Centre, National Institute of Advanced Industrial Science and Technology 2-2-9 Machiikedai, Koriyama Fukushima 963-0298 Japan yasu-kobayashi@aist.go.jp; b Department of Chemical Science and Engineering, National Institute of Technology, Tokyo College 1220-2 Kunugida, Hachioji Tokyo 193-0997 Japan

## Abstract

A molten salt method was used to prepare CrMnFeNi alloy nanopowder passivated by TiO_*x*_–ZrO_*y*_ surface shell with a high specific surface area (23 m^2^ g^−1^) from the oxide precursors. Analyses by scanning electron microscopy/transmission electron microscopy with energy-dispersive X-ray spectroscopy and X-ray photoelectron spectroscopy revealed the formation of an alloyed Cr–Mn–Fe–Ni-rich core surrounded by an oxide surface shell with a Ti/Zr-rich composition, confirming the formation of TiO_*x*_–ZrO_*y*_/CrMnFeNi nanopowder. It was speculated that the CrMnFeNi alloy nanoparticles were preferentially formed from the constituent metals by a faster reduction of any oxides of Cr, Mn, Fe, and Ni and a subsequent alloying with Ti and Zr could hardly occur due to the high thermodynamic stability of CrMnFeNi alloy. A Ni-loaded TiO_*x*_–ZrO_*y*_/CrMnFeNi catalyst exhibited superior catalytic performance to common Ni-loaded TiO_2_ and ZrO_2_ in the liquid-phase hydrogenation of *p*-nitrophenol at room temperature. The enhancement could have originated from an excellent electrical property of CrMnFeNi alloy, promoting the formation of active metallic nickel on the surface during the reaction. Leaching amounts of the constituent elements of Ti–Zr–Cr–Mn–Fe–Ni and loaded Ni was very little in the reaction solution after the reaction; the results confirmed that the prepared CrMnFeNi alloy nanopowder was very stable due to the protection of the Ti/Zr-rich oxide shell. Thus, the potential application of the alloyed powder used as catalyst support was demonstrated.

## Abstract 2

The enhancement of liquid-phase hydrogenation by NaBH_4_ over Ni-loaded CrMnFeNi alloy nanoparticles passivated by TiO_*x*_–ZrO_*y*_.

## Graphical abstract

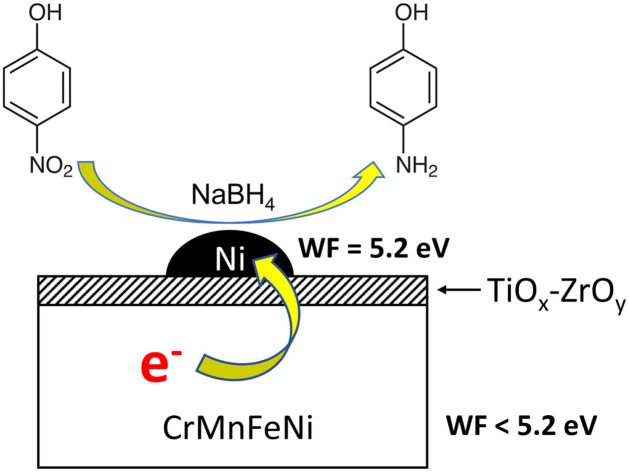

## Introduction

Catalyst supports are one of the very important materials to obtain a high catalytic performance in various energy and environmental application fields due to a good dispersion of loaded active species.^1^ The supports should be stable/inactive in reaction conditions and have high specific surface areas. Therefore, high specific surface area oxides and carbonaceous materials, as they meet these requirements, are commonly used as a first choice of catalyst supports. Contrary to the conventional approach, alloyed catalyst supports have gained considerable attention due to their superior heat transfer performance. The iron–chromium–aluminum alloy, such as FeCrAl alloy and Kanthal, is one of the most studied alloys available for industrial catalyst supports.^2^ The FeCrAl-based structured catalysts show remarkable thermal conductivity minimizing hot spots and thermal gradients during reactions. The aluminum oxide surface shell formed by an initial oxidation treatment of the alloys protects from further inner oxidation, thus allowing FeCrAl alloy support to be available under high-temperature reaction conditions. To date, FeCrAl alloy supports have been used in several catalytic reactions involving methane oxidation,^3–8^ selective catalytic reduction (SCR) of NO_*x*_,^9^ CO oxidation,^10^ methanation,^11^ reforming,^12–16^ and Fischer–Tropsch synthesis.^17^ In our previous work, we used an intermetallic compound Ti_6_Si_7_Ni_16_ with a high specific surface area (37.5 m^2^ g^−1^) as catalyst support in CO methanation.^18^ Similar to the FeCrAl alloy support, it was demonstrated by XPS measurements that the surface of Ti_6_Si_7_Ni_16_ was passivated by a stable TiO_*x*_-SiO_*y*_ oxidation shell to give Ti_6_Si_7_Ni_16_ nanoparticles handled in air while maintaining the high specific surface area. The results showed a tremendously high catalytic performance because of a superior electron-releasing property of Ti_6_Si_7_Ni_16_ to a loaded Ni species. The result opened a new application field for core-shelled alloys as catalyst support possessing the alloy's excellent thermal and electrical properties.

In this study, we prepared a CrMnFeNi alloy nanopowder passivated by a TiO_*x*_–ZrO_*y*_ surface shell, expressed as TiO_*x*_–ZrO_*y*_/CrMnFeNi, using a molten salt synthesis method. An equimolar CrMnFeNi solid solution alloy is known as one kind of high-entropy alloy (HEAs),^19–21^ piquing the interest of researchers these days because of their superior physical/chemical properties.^22–25^ Since the work functions of Cr (4.5 eV), Mn (4.1 eV), and Fe (4.7 eV) are lower than Ni (5.2 eV), the CrMnFeNi alloy can have a lower work function than Ni. Therefore, based upon the same concept of Ti_6_Si_7_Ni_16_ catalyst support possessing a low work function (4.5 eV) to release electrons to a loaded Ni active species, the availability of the CrMnFeNi alloy used as catalyst support for Ni-loaded catalysts was expected. In order to inactivate the CrMnFeNi alloy, it was attempted to cover the surface with common oxide supports of TiO_2_ and ZrO_2_. The core-shelled alloy powder was prepared by reducing the oxide precursor containing Cr, Mn, Fe, Ni, Ti, and Zr with CaH_2_ in molten LiCl at 800 °C. Finally, the availability of the prepared alloy powder as a catalyst support was investigated in the liquid-phase hydrogenation of *p*-nitrophenol as a model reaction.

## Experimental

### Preparation of TiO_*x*_–ZrO_*y*_/CrMnFeNi nanopowder

CrMnFeNi alloy nanopowder passivated by TiO_*x*_–ZrO_*y*_ surface shell was prepared by reducing oxide precursors in a molten LiCl–CaH_2_ mixture at 600 °C or 800 °C.^26^ The oxide precursor was prepared using the following citric acid method. First, TiO_2_ nanopowder (99.5%, JRC-TIO-17) and any salts of the other constituent elements of ZrO(NO_3_)_2_·2H_2_O (97%, Wako Pure Chem. Corp.), Cr(NO_3_)_3_·9H_2_O (98%, Nacalai Tesque, Inc.), Mn(NO_3_)_2_·6H_2_O (98%, Wako Pure Chem. Corp.), Fe(NO_3_)_3_·9H_2_O (99%, Wako Pure Chem. Corp.), Ni(NO_3_)_2_·6H_2_O (99.9%, Wako Pure Chem. Corp.) were suspended/dissolved in distilled water with an equimolar ratio of Ti/Zr/Cr/Mn/Fe/Ni = 1/1/1/1/1/1. Then, citric acid was added to the solution with a molar ratio of salts/citric acid = 1/1.2. After the components were sufficiently mixed, the resulting solution was evaporated on a hotplate at 110 °C overnight. The dried powder was preliminarily heated at 250 °C in the air for 2 h and then gently mixed in a mortar to obtain a homogeneous powder. Finally, the powder was heated at 500 °C in the air for 2 h to obtain the oxide precursor, referred to as TiZrCrMnFeNi(Pre). Following that, the oxide precursor, CaH_2_ (JUNSEI Chem. Co.), and LiCl (Wako Pure Chem. Corp.) were mixed in a mortar with a weight ratio of 2/6/3 of Pre/CaH_2_/LiCl, respectively. The mixed powder was placed in a stainless-steel container filled with N_2_ gas and heated at 600 °C or 800 °C for 2 h. Finally, the reduced precursor was crushed in a mortar and rinsed several times with 0.1 M NH_4_Cl aqueous solution and distilled water. The dried final products previously heated at 600 °C and 800 °C were named as TiZrCrMnFeNi(600) and TiZrCrMnFeNi(800), respectively.

### Characterization

The crystal structure was examined using X-ray diffraction (XRD, MiniFlex 600, Rigaku) with CuK_α_ radiation at 40 kV and 15 mA. The porosity was investigated using N_2_ adsorption at −196 °C (BELLSORP mini-II, Microtrac-BEL). The sample was pretreated at 200 °C for 30 min under a vacuum before the measurement. Scanning electron microscopy (SEM, JSM-7400F, JEOL Ltd) and transmission electron microscopy (TEM, a Tecnai Osiris, FEI system) were used to observe the morphology with energy dispersive X-ray spectrometry (EDX) for elemental analysis. The EDX (EDX-8000, Shimadzu Corp.) was used to determine the constituent elemental molar ratios in the prepared sample powders. The chemical states and composition of the prepared sample's surface were identified using X-ray photoelectron spectroscopy (XPS) (PHI X-tool, ULVAC-PHI) operated with AlK_α_ radiation. The chemical shifts were calibrated by fixing the C 1s peak of the surface carbonaceous contaminants to 284.8 eV. The leaching amounts of constituent elements in the used catalysts after the reactions were analyzed by inductively coupled plasma atomic emission spectroscopy (ICP–AES) (PS7800, Hitachi Ltd.). Oxygen content in TiZrCrMnFeNi(800) over a macroscopic range was measured using elemental analysis (TCH-600, LECO corporation). Before the measurement, the sample powder (10 mg) was preheated at 150 °C for 2 h under an Ar flow (400 mL min^−1^). The measurements were conducted three times to confirm the validity of the obtained oxygen contents.

### Catalytic test

The catalytic reactions were performed in 20 mL glass bottles following the previously reported procedures.^27^ A common impregnation method was used to prepare Ni-loaded TiZrCrMnFeNi(800) catalyst. For comparison, Ni catalysts loaded on commercial nanopowders of TiO_2_ (Evonik Industries AG, 50 m^2^ g^−1^) and ZrO_2_ (Sigma-Aldrich Co. LLC, 25 m^2^ g^−1^) were also prepared in a similar manner. First, Ni(NO_3_)_2_·6H_2_O was dissolved in distilled water. Then, TiZrCrMnFeNi(800), TiO_2_, or ZrO_2_ powder was suspended in the solution with a 10 wt% Ni loading amount. The solution was then dried at 110 °C to obtain the precursor. Finally, the precursor was heated in the reactor at 500 °C for 1 h under Ar flow to obtain the Ni-loaded catalyst, denoted by Ni/TiZrCrMnFeNi(800), Ni/TiO_2_, or Ni/ZrO_2_. In the catalytic tests, 1 mL of *p*-nitrophenol (4-NP) solution (14 mM) was added to a bottle containing 10 mg of catalyst powder, 1 mL of NaBH_4_ solution (0.42 M), and 7 mL of distilled water as the solvent. To satisfy the first-order reaction kinetics, the initial concentration of NaBH_4_ (0.047 M) was 30 times higher than that of 4-NP (1.6 mM). The reactions were conducted under stirring at 50 °C for 30 min. An aluminum heat sink on a hotplate was used to maintain a constant solution temperature. A small aliquot (100 μL) of the reaction solution was taken to determine the concentration changes during the reaction. The conversion of 4-NP to *p*-aminophenol was monitored using an ultraviolet-visible spectrometer (Shimadzu, UV-1280), and the respective absorbance changes at 401 and 315 nm.

## Results and discussion

### Preparation of TiO_*x*_–ZrO_*y*_/CrMnFeNi nanopowder

**Fig. 1:**
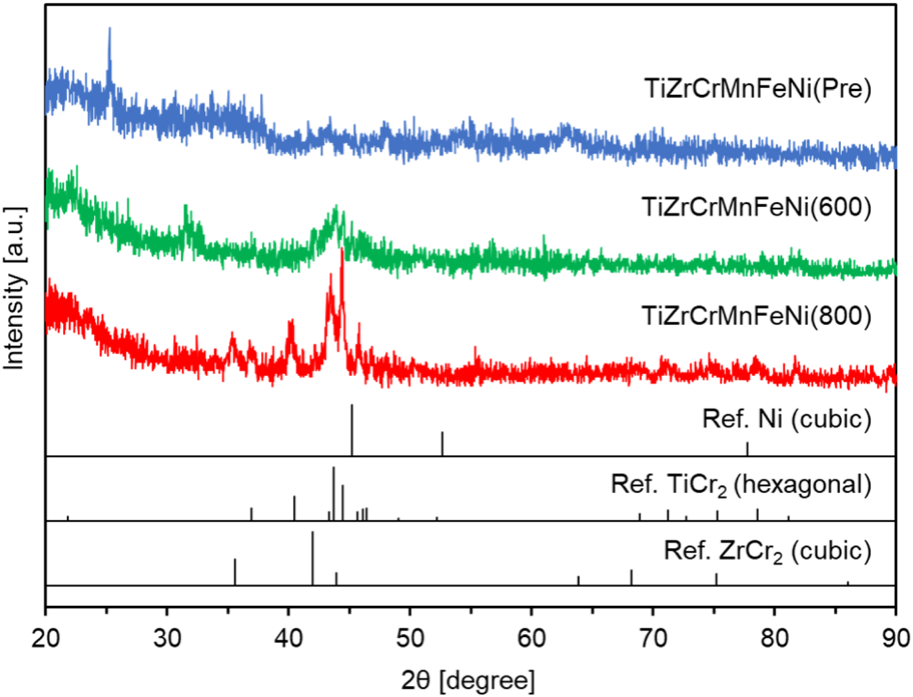
XRD patterns for TiZrCrMnFeNi(Pre), TiZrCrMnFeNi(600), and TiZrCrMnFeNi(800) with references of Ni, TiCr_2_ and ZrCr_2_.

Fig. 1 shows the XRD patterns of TiZrCrMnFeNi(Pre), TiZrCrMnFeNi(600), and TiZrCrMnFeNi(800). For TiZrCrMnFeNi (Pre), some unknown small peaks were observed, indicating the formation of amorphous mixed phases of any oxides. For TiZrCrMnFeNi(600), any peaks observed in TiZrCrMnFeNi(Pre) disappeared and instead broad peaks around 40° were mainly observed. These peaks hardly identified any phases, probably due to insufficient reduction/alloying. Therefore, the reduction temperature was then increased to 800 °C to obtain a high crystallite sample. For TiZrCrMnFeNi(800), clearer peaks were observed around 30–50°. According to the previous works,^19,20^ CrMnFeNi HEA has a face-centered cubic (FCC) structure, which corresponds to the crystal structure of nickel metal. After careful examination of the peak assignment results (Fig. S1†), the peaks assigned to FCC Ni were not observed in TiZrCrMnFeNi(800). They were partially assigned to a TiCr_2_-type hexagonal structure and a ZrCr_2_-type cubic structure. The crystal system for AB_2_ (A = Ti or Zr, B = Cr, Mn, Fe, or Ni) is hexagonal or cubic, as summarized in Table S1† using an open-access database.^28^ Therefore, our results suggested that TiZrCrMnFeNi(800) was mainly composed of mixed phases of hexagonal and cubic structures. According to the nitrogen adsorption for TiZrCrMnFeNi(800), the obtained BET-specific surface area was 22.5 m^2^ g^−1^ (Table 1). The value was smaller than 37.5 m^2^ g^−1^ of the intermetallic compound Ti_6_Si_7_Ni_16_ prepared at 600 °C in a similar manner.^18^ The high reduction temperature of 800 °C could cause the smaller surface area in TiZrCrMnFeNi(800), but it was high enough for the application of catalyst support. In summary, the TiCr_2_-type hexagonal TiZrCrMnFeNi powder with a high specific surface area was obtained by reducing the oxide precursor at 800 °C.

**Table 1:** BET surface areas (SA), and elemental molar ratios measured using EDX-8000, XPS, SEM-EDX, and TEM-EDX^a^

Sample	SA [m^2^ g^−1^]	Elemental molar ratio [mol%]								
		Method^b^	Position	Ti	Zr	Cr	Mn	Fe	Ni	Average
TiZrCrMnFeNi(Pre)	—	EDX-8000	Overall	11.4	26.9	13.5	15.6	16.2	16.4	—
		XPS	#1	10.2	20.7	20.9	20.2	14.5	13.5	Ti/Zr/Cr/Mn/Fe/Ni = 13/19/18/21/16/12
			#2	10.4	19.1	15.1	20.3	21.3	13.8	
			#3	19.4	17.8	18.8	21.8	13.0	9.2	
TiZrCrMnFeNi(800)	22.5	EDX-8000	Overall	11.9	25.8	13.8	15.6	16.6	16.2	—
		XPS	#1	30.5	42.9	7.6	7.2	6.2	5.6	Ti/Zr/Cr/Mn/Fe/Ni = 29/40/11/8/8/4
			#2	30.8	39.4	16.1	6.0	7.7	0	
			#3	25.4	37.3	9.4	11.4	9.1	7.4	
		SEM-EDX	Overall	13.9	42.0	11.5	10.5	11.3	10.7	Ti/Zr/Cr/Mn/Fe/Ni = 13/42/11/11/12/12
			#1	12.4	67.6	5.3	4.8	5.2	4.8	
			#2	14.9	32.7	14.0	12.0	13.2	13.1	
			#3	11.1	24.4	15.0	15.7	16.5	17.3	
		TEM-EDX	#1	10.3	7.3	63.5	7.3	10.1	1.4	Ti/Zr/Cr/Mn/Fe/Ni = 19/35/14/10/10/12
			#2	1.8	0.9	14.9	26.9	27.8	27.7	
			#3	60.0	9.9	24.9	1.7	3.0	0.5	
			#4	3.8	2.5	12.8	26.9	26.8	27.1	
			#5	35.9	22.4	30.2	3.5	6.3	1.7	
			#6	4.5	4.6	69.7	8.3	11.5	1.3	
			#7	2.4	3.8	8.1	32.2	21.2	32.3	
			#8	4.2	6.7	21.7	22.0	27.8	17.6	
			#9	4.8	6.8	10.2	28.2	22.3	27.7	
			#10	10.5	88.1	0.2	0.2	0.5	0.6	
			#11	23.3	19.2	4.4	17.4	17.4	18.3	
			#12	86.7	11.6	0.4	0.4	0.5	0.5	
			#13	10.6	87.8	0.3	0.3	0.6	0.3	
			#14	7.6	39.0	0.7	3.2	4.0	45.5	
			#15	14.5	22.5	11.2	16.4	16.9	18.6	
			#16	49.9	41.8	0.9	1.7	2.5	3.2	
			#17	3.2	29.8	54.4	7.8	3.8	1.1	
			#18	17.0	19.7	9.9	18.8	19.1	15.4	
			#19	28.8	69.8	0.4	0.3	0.6	0.2	
			#20	7.5	38.1	0.7	3.2	4.3	46.2	
			#21	11.4	22.6	27.3	15.4	13.8	9.5	
			#22	21.2	77.4	0.2	0.6	0.6	0	
			#23	12.4	86.1	0.3	0.2	0.5	0.4	
			#24	13.8	32.1	6.7	16.1	16.4	15.0	
			#25	25.1	73.2	0.3	0.5	0.8	0.1	
			#26	24.1	73.7	0.4	0.5	0.9	0.3	

a Converted elemental weight ratios for XPS, SEM-EDX, and TEM-EDX are described in Table S2.

b Elemental analyses for XPS, SEM-EDX, and TEM-EDX were separately conducted at 3, 3, and 26 different positions, respectively.

The constituent elemental ratios for TiZrCrMnFeNi(Pre) and TiZrCrMnFeNi(800) were measured over a macroscopic range using the EDX-8000. The obtained EDX spectra are shown in Fig. S2.† The obtained molar ratios for Ti, Zr, Cr, Mn, Fe, and Ni are summarized in Table 1. All the constituent elements were identified. The molar ratios of Ti/Zr/Cr/Mn/Fe/Ni were 11.4/26.9/13.5/15.6/16.2/16.4 and 11.9/25.8/13.8/15.6/16.6/16.2 for TiZrCrMnFeNi(Pre) and TiZrCrMnFeNi(800), respectively. In both samples, the same ratios were reasonably obtained for all elements except Zr. The same results of excess Zr were also observed in SEM–/TEM–EDX, which is discussed as follows. Therefore, some Zr species could be localized in the samples.

**Fig. 2:**
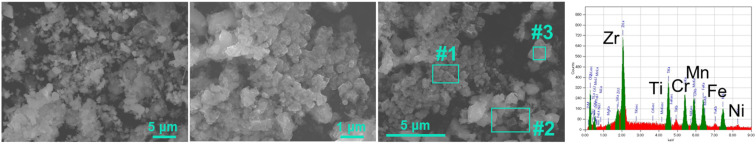
SEM images for TiZrCrMnFeNi(800) and the EDX spectrum of the overall view for a left-down image. The corresponding EDX spectra for positions #1–#3 are shown in Fig. S3.†

Furthermore, to examine the constituent elemental ratio and distribution over a microscopic range, SEM-EDX spectra were obtained for TiZrCrMnFeNi(800). The SEM images and an EDX spectrum of the overall view for the left-side image are shown in Fig. 2. The elemental analyses by EDX were performed at three different positions shown in Fig. 2 and their spectra are shown in Fig. S3.† Average and separate molar ratios of Ti/Zr/Cr/Mn/Fe/Ni for positions #1–#3 are summarized in Table 1. The images show that nanosized morphologies of <100 nm were observed, indicating the origin of the high specific surface area in TiZrCrMnFeNi(800). In the EDX experiments, some impurity elements were detected except for the constituent elements of Ti, Zr, Cr, Mn, Fe, and Ni. Similar to the results of EDX-8000, excess ratios of Zr were detected at different positions, but the molar ratios of the other constituent elements were reasonably equal, with an average molar ratio of Ti/Zr/Cr/Mn/Fe/Ni = 13.1/41.7/11.4/10.8/11.6/11.5.

**Fig. 3:**
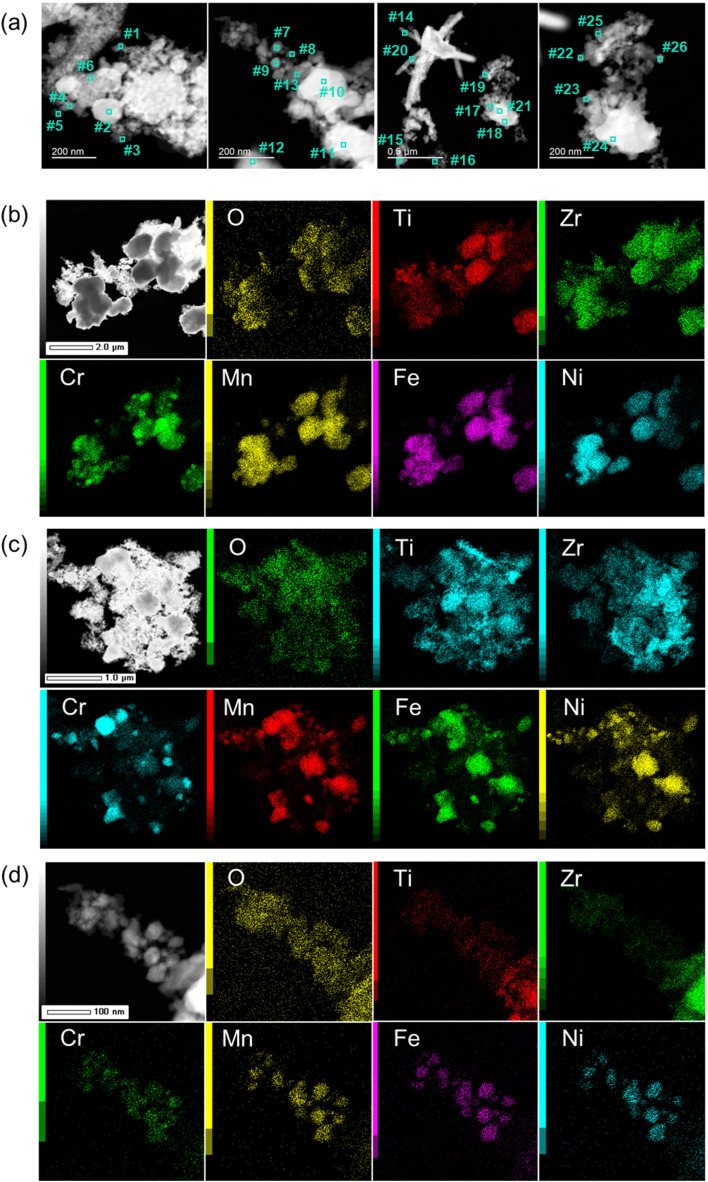
(a) TEM images and (b–d) the constituent elemental mappings for TiZrCrMnFeNi(800) at three different positions. The molar ratios of Ti/Zr/Cr/Mn/Fe/Ni for positions #1–#26 are summarized in Table 1.

To examine the finer morphology with elemental analysis, TEM-EDX was performed for TiZrCrMnFeNi(800) (Fig. 3 and S4†). The elemental analyses were performed using EDX at the 26 different positions shown in Fig. 3(a). The detected average and separate elemental molar ratios are summarized in Table 1. Nanoscale particles were observed in the TEM images. The constituent molar ratios of Ti, Zr, Cr, Mn, Fe, and Ni were significantly different from each other at different positions observed in such nanoscale range, but the average values were reasonably the same except for excess Zr. The detected positions for Cr, Mn, Fe, and Ni nearly overlapped in the elemental mappings (Fig. 3(b)–(d)). Especially for Fig. 3(d), it was clearly observed that the elements of Cr, Mn, Fe, and Ni were homogeneously distributed on the small nanoparticles of <50 nm. The results indicated good alloying among the elements in a nanoscale range. However, Ti and Zr were more widely dispersed than the other elements in the mapping images. Since the same wide distribution of oxygen was also detected in the mapping, some portions of Ti and Zr could be oxides rather than alloys.

**Fig. 4:**
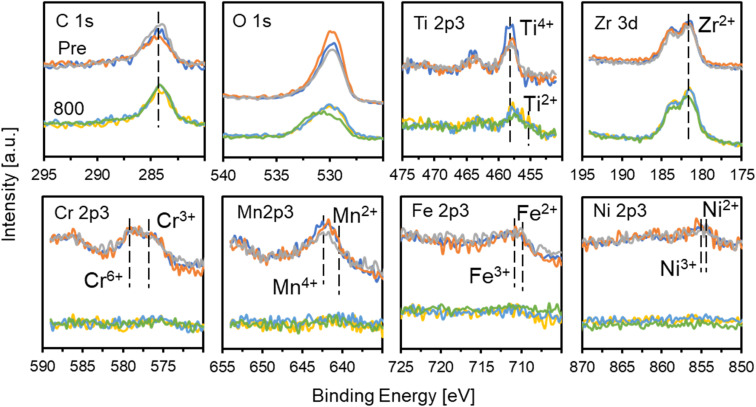
XPS spectra for TiZrCrMnFeNi(Pre) (top) and TiZrCrMnFeNi(800) (down). Each data was measured at 3 different positions.

To examine the surface chemical states, XPS measurements were conducted for TiZrCrMnFeNi(Pre) and TiZrCrMnFeNi(800). The analyses were performed at 3 different positions for each sample to ensure the measurement errors. The obtained spectra for C 1s, O 1s, Ti 2p3, Zr 3d, Cr 2p3, Mn 2p3, Fe 2p3, and Ni 2p3 orbitals and the surface molar ratios of Ti/Zr/Cr/Mn/Fe/Ni are described in Fig. 4 and Table 1, respectively. Clear signals for O 1s orbitals were observed in both TiZrCrMnFeNi(Pre) and TiZrCrMnFeNi(800) samples. The results indicated that most of the surfaces were in the form of oxides. For Ti 2p3, Zr 3d, Cr 2p3, Mn 2p3, Fe 2p3, and Ni 2p3 orbitals in TiZrCrMnFeNi(Pre), signals that were assignable to the oxidation states of Ti(+4),^29,30^ Zr(+2),^31^ Cr(+6)/Cr(+3),^32^ Mn(+4)/Mn(+2),^33^ Fe(+3)/Fe(+2),^34^ and Ni(+3)/Ni(+2)^35^ were observed. However, for those orbitals in TiZrCrMnFeNi(800), signals to Ti(+4)/Ti(+2)^29,30^ and Zr(+2)^31^ were observed, while very small signals of Cr, Mn, Fe, and Ni were observed. These results showed that any elements of Cr, Mn, Fe, and Ni were little exposed on the surface, and instead the surface was mainly composed of Ti-/Zr-oxides. Considering the results of XRD, SEM-/TEM-EDX, and XPS, the prepared TiZrCrMnFeNi(800) powder had the following core–shell structure, the core of CrMnFeNi alloy and the shell of Ti-/Zr-oxides, expressed as TiO_*x*_–ZrO_*y*_/CrMnFeNi.

The core–shell alloy powder was prepared by reducing the oxide precursor containing Cr, Mn, Fe, Ni, Ti, and Zr with CaH_2_ in a molten LiCl at 800 °C. In the molten salt synthesis, hydride ions (H^−^) released due to the dissolution of CaH_2_ into molten LiCl work as a main reducing agent (eqn (1)).

1 CaH_2_ → Ca^2+^ + 2H^−^

In addition, CaH_2_ thermally decomposes to form metallic Ca above 600 °C (eqn (2)).

2 CaH_2_ → Ca + H_2_

The formed H^−^ and metallic Ca have very strong reduction capacities with reduction potentials of −2.2 V (*vs.* standard hydrogen electrode) and −2.9 V, respectively. Since the potentials of these reductants are lower than those of Cr (−0.7 V), Mn (−1.2 V), Fe (−0.4 V), Ni (−0.3 V), Ti (−1.4 V), and Zr (−1.5 V), the Cr–Mn–Fe–Ni–Ti–Zr oxides are theoretically reduced by the H^−^ and metallic Ca in molten LiCl above 600 °C. However, the rates of reduction could be different from each other due to the difference in the reduction potentials and maybe a faster reduction could occur in the following order of Ni > Fe > Cr > Mn > Ti > Zr. So, it is speculated that the formed metallic Ni could alloy with metallic Fe first, and next the Ni–Fe alloy similarly react with metallic Cr. Since equimolar CrMnFeNi is a stable HEA,^19–21^ it is considered that further alloying of the formed CrMnFeNi with Ti and Zr could hardly occur and the unalloyed Ti and Zr remained around the CrMnFeNi alloy particles. In the subsequent washing treatments, the metallic Ti and Zr were oxidized to be in an oxide form surrounding the CrMnFeNi alloy particles. Thus, the CrMnFeNi alloy particles with the TiO_*x*_–ZrO_*y*_ surface shell were prepared by taking advantage of the difference in the reduction rates of constituent elemental oxides.

Oxygen content in TiZrCrMnFeNi(800) over a macroscopic range was measured by LECO TCH-600 three times. Each oxygen content and the average content are described in Tables S3† and 2, respectively. In comparison with the oxygen contents measured by SEM-EDX and TEM-EDX over a microscopic range (Table 2), the value obtained by LECO TCH-600 was reasonably close to the values obtained by the microscopic analysis methods of SEM-EDX and TEM-EDX. On the other hand, the value measured by XPS, which is a surface-sensitive method was significantly different from the other values. The results indicated that the oxygen contained in the sample was mostly located on the surface, confirming the formation of oxide passivation. In the preparation process, the released hydride ions (H^−^) from CaH_2_ reducing agent in molten LiCl could reduce TiZrCrMnFeNi(Pre). An excess amount of CaH_2_ was actually added to the mixture before the reduction process. Thus, it is speculated that the incomplete reduction in TiZrCrMnFeNi(800) could be due to the insufficient diffusion of H^−^ in molten LiCl. The issue might be solved by improving the stirring efficiency of the suspension of precursors and CaH_2_ in molten LiCl to promote the reduction reaction.

**Table 2:** Average weight percentages of oxygen contents measured by XPS, SEM-EDX, TEM-EDX, and LECO TCH-600 for TiZrCrMnFeNi(800)

Sample	Method	Oxygen content [wt%]
TiZrCrMnFeNi(800)	XPS	38.1
	SEM-EDX	9.5
	TEM-EDX	4.2
	LECO TCH-600	4.4

### Catalytic test

**Fig. 5:**
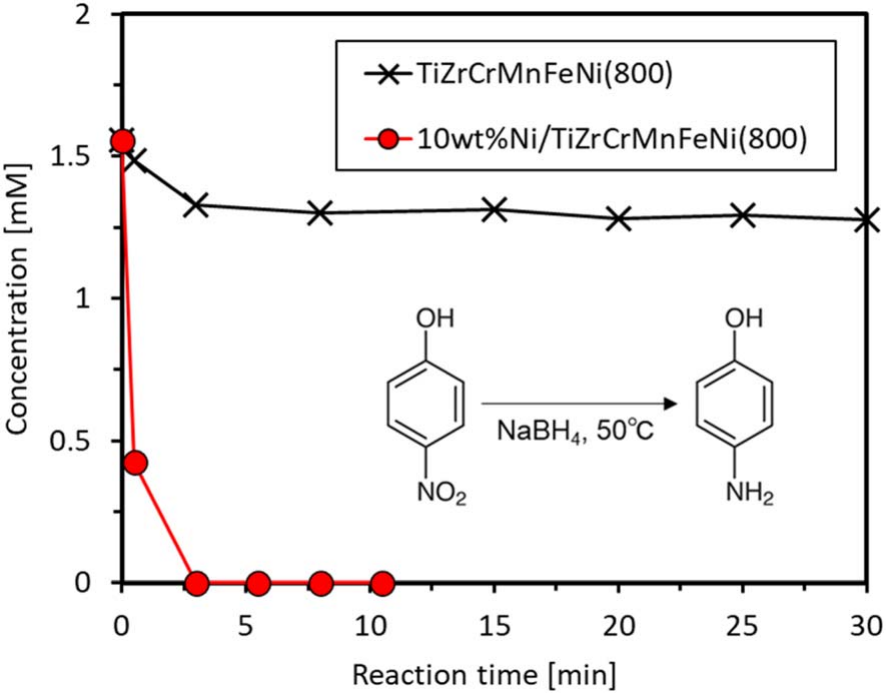
Changes in 4-NP concentration *versus* time at 50 °C for TiZrCrMnFeNi(800) and Ni/TiZrCrMnFeNi(800).

First, the catalytic hydrogenation of 4-NP by NaBH_4_ was conducted at 50 °C. The concentration changes of 4-NP during the reactions catalyzed by TiZrCrMnFeNi(800) and Ni/TiZrCrMnFeNi(800) are shown in Fig. 5. The concentration slightly decreased from 1.6 to 1.3 mM for TiZrCrMnFeNi(800) in an initial state of 3 min but did not change further during the 30 min reaction time. The initial slight decrease could be due to the adsorption of 4-NP on the vast surface of TiZrCrMnFeNi(800), and the subsequent catalytic reaction did not proceed on the saturated surface. Since the hydrogenation was catalyzed by any metals and alloys of Cr, Mn, Fe, and Ni,^27^ the noncatalyzed result also verified that metals were little exposed on the surface of TiZrCrMnFeNi(800). Furthermore, nickel-loaded TiZrCrMnFeNi(800) was tested under the same reaction conditions. The concentration quickly decreased and reached zero in 3 min. The results indicated that 4-NP was nicely catalyzed by the Ni species loaded on TiZrCrMnFeNi(800). The ICP measurements were used to investigate the leaching amounts of any constituent elements of the used catalysts into the solution after the reaction. Pretty small amounts of <0.01 wt% were detected for all elements, as shown in the three times results in Table 3. Thus, TiZrCrMnFeNi(800) was inactive and stable in the reductive reaction conditions due to the protection of Ti/Zr-rich oxide shell, indicating that the prepared TiZrCrMnFeNi(800) can be nicely used as a catalyst support.

**Table 3:** Leaching amounts of constituent elements after reactions at 50 °C with TiZrCrMnFeNi(800) and Ni/TiZrCrMnFeNi(800) by ICP measurements

Sample	Leaching amounts after reaction [×10^−4^ wt%]						
	No.	Ti	Zr	Cr	Mn	Fe	Ni
TiZrCrMnFeNi (800)	1	18.0	3.4	18.0	1.1	10.1	22.5
	2	1.1	7.9	16.9	1.1	11.3	24.8
	3	2.3	6.8	13.5	1.1	12.4	22.5
	Avg.	7.1	6.0	16.1	1.1	11.3	23.3
Ni/TiZrCrMnFeNi (800)	1	1.1	6.8	14.6	79.9	10.1	16.9
	2	1.1	5.6	11.3	77.6	7.9	20.3
	3	1.1	6.8	14.6	81.0	10.1	19.1
	Avg.	1.1	6.4	13.5	79.5	9.4	18.8

**Fig. 6:**
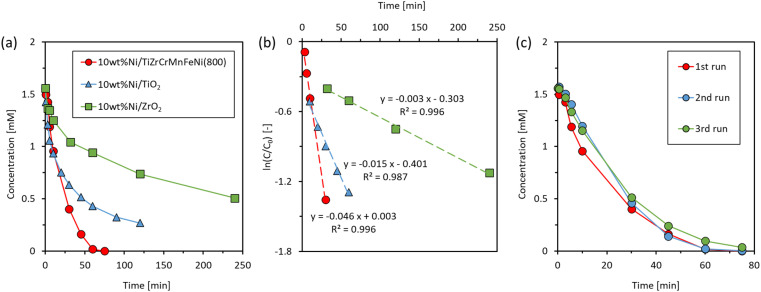
(a) Changes in 4-NP concentration (*C*) *versus* time, (b) the plots of ln(*C*/*C*_0_) *versus* time for Ni/TiZrCrMnFeNi(800), Ni/TiO_2_ and Ni/ZrO_2_, and (c) the repeatability for Ni/TiZrCrMnFeNi(800) at 25 °C.

**Fig. 7:**
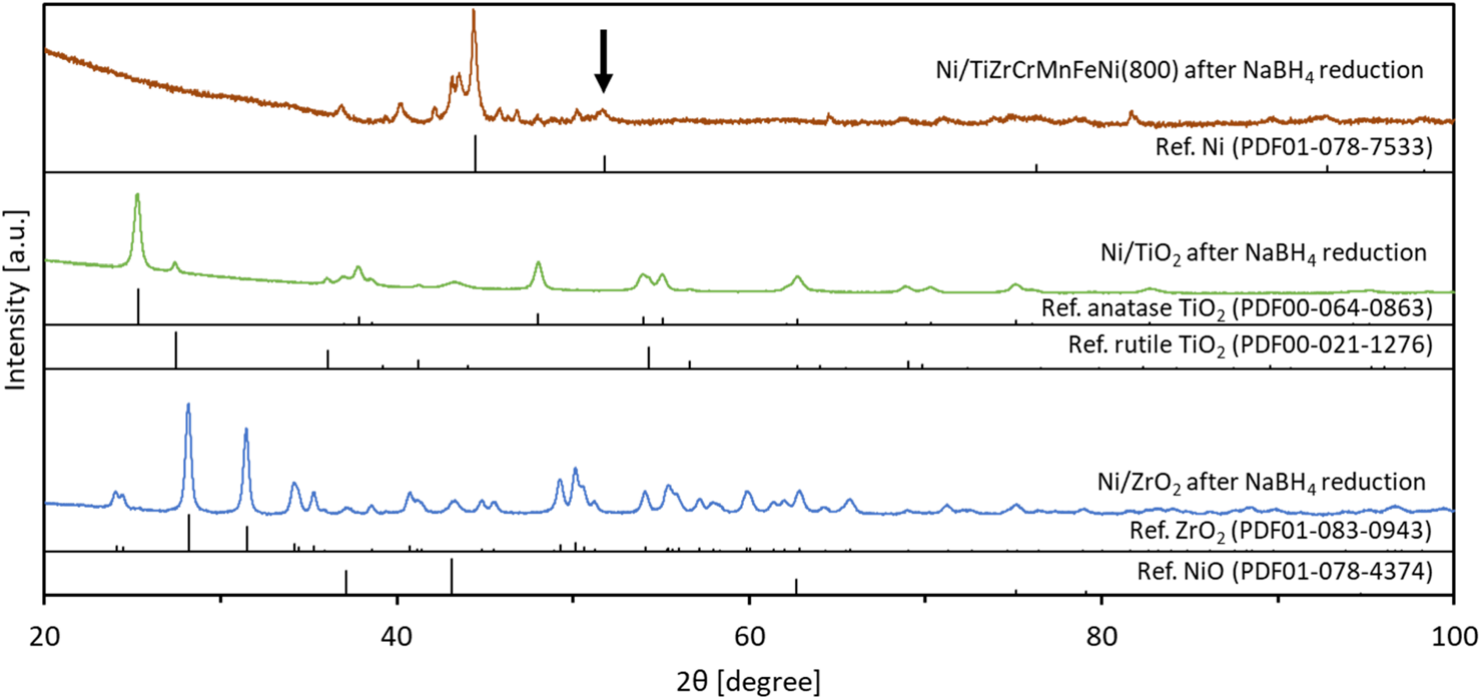
XRD patterns for Ni/TiZrCrMnFeNi(800), Ni/TiO_2_, and Ni/ZrO_2_ after being reduced by NaBH_4_ at 25 °C for 30 min with references of Ni, NiO, TiO_2_, and ZrO_2_. An arrow indicates a position of 51.8° corresponding to a signal for a (2 0 0) plane of metallic Ni. An enlarged pattern for Ni/TiZrCrMnFeNi(800) after the reduction is given in Fig. S5.†

Next, the catalytic hydrogenation activities were compared among Ni/TiZrCrMnFeNi(800), Ni/TiO_2_, and Ni/ZrO_2_. For Ni/TiZrCrMnFeNi(800), Ni was actually loaded on the Ti/Zr-rich oxide shell and the supported catalyst can be expressed as Ni/TiO_*x*_–ZrO_*y*_/CrMnFeNi. The specific surface areas of TiZrCrMnFeNi(800), TiO_2_, and ZrO_2_ were 22.5 m^2^ g^−1^, 50 m^2^ g^−1^, and 25 m^2^ g^−1^, respectively. Therefore, it was a good comparison among the Ni/TiO_*x*_–ZrO_*y*_/CrMnFeNi, Ni/TiO_2_, and Ni/ZrO_2_ in order to examine the effect of alloy properties in TiZrCrMnFeNi(800) on the catalytic activity in the loaded catalyst. Fig. 6(a) shows the concentration changes of 4-NP as a function of time at 25 °C. The concentrations were slowly decreased with Ni/TiO_2_ and Ni/ZrO_2_, whereas the concentration was quickly decreased with Ni/TiZrCrMnFeNi(800) and reached zero in 60 min. Thus, the active nickel species loaded on TiZrCrMnFeNi(800) catalyzed the hydrogenation of 4-NP more effectively than those loaded on the common catalyst supports of TiO_2_ and ZrO_2_. In order to estimate the active nickel species during the reaction, XRD measurements were conducted for Ni/TiZrCrMnFeNi(800), Ni/TiO_2_, and Ni/ZrO_2_ after NaBH_4_ reduction. The samples were previously stirred in a NaBH_4_ solution of the same concentration as the reaction solution at 25 °C for 30 min, and then the samples were filtered and dried to be used for the measurements. Fig. 7 indicates the XRD patterns for Ni/TiZrCrMnFeNi(800), Ni/TiO_2_, and Ni/ZrO_2_ after the NaBH_4_ reduction with references of Ni, NiO, TiO_2_, and ZrO_2_. An enlarged pattern for the reduced Ni/TiZrCrMnFeNi(800) is given in Fig. S5.† For the reduced Ni/TiZrCrMnFeNi(800), a peak assigned to a (2 0 0) plane of metallic Ni was newly observed at 51.8° after the reduction, suggesting that the active nickel species during the reaction was metallic nickel for Ni/TiZrCrMnFeNi(800). On the other hand, for the reduced Ni/TiO_2_ and Ni/ZrO_2_, peaks of metallic Ni were not observed and instead peaks of NiO were observed other than the peaks assigned to TiO_2_ and ZrO_2_, respectively. The results indicated that the loaded Ni species on TiO_2_ and ZrO_2_ could not be reduced during the reaction and the actual active species were nickel oxides. Apart from the pure oxide supports, the core-alloyed support of TiO_*x*_–ZrO_*y*_/CrMnFeNi could have superior properties of heat and electrical conductivities. As reported in the previous works,^2,18^ these support properties sometimes work to promote catalytic activities. As mentioned in the introduction part, the CrMnFeNi alloy can have a lower work function than Ni because the work functions of Cr, Mn, and Fe are lower than that of Ni. It means that an electron transfer from the CrMnFeNi alloy support to the loaded Ni species can occur, probably throughout the surface oxide shell during the reaction. Thus, it was speculated on a basis of the results of XRD experiments showing the formation of different Ni species on the alloyed support and oxide supports that the electron transfer from CrMnFeNi alloy support to the loaded Ni species could help the appearance of active metallic Ni in Ni/TiZrCrMnFeNi(800). Since metallic Ni has a higher hydrogenation activity than NiO,^36^ Ni/TiZrCrMnFeNi(800) loaded with active metallic Ni could show a higher hydrogenation performance than Ni/TiO_2_ and Ni/ZrO_2_ loaded with less active NiO.

The rate constants (*k*) for Ni/TiZrCrMnFeNi(800), Ni/TiO_2_, and Ni/ZrO_2_ were obtained from plots of ln(C/C_0_) *versus* time shown in Fig. 6(b). Good linearities were given for all the samples, and the obtained *k* values of this study and previous studies with nickel-based catalysts are summarized in Table 4. In order to compare the rate constants among the catalysts with different loading amounts of nickel, the constants (*k*′) normalized by the loading amounts of nickel are also indicated in Table 4. Since the reaction conditions differed, a quantitative comparison with previous works is difficult. Our result of Ni/TiZrCrMnFeNi(800) was reasonably comparable with the previous works with unsupported Ni catalysts. It may be suggested that our alloy catalyst has a similar metallic property to the unsupported nickel catalysts. Finally, the repeatability and stability of Ni/TiZrCrMnFeNi(800) were examined by evaluating the catalytic performance in repeated experiments. Fig. 6(c) shows the changes in the concentration of 4-NP *versus* time for three continuous experiments with an identical catalyst of Ni/TiZrCrMnFeNi(800) at 25 °C. in all experiments, the concentrations nearly reached zero. In the second and third runs, the reaction rates were slightly slower than the rate in the first run, but the difference was small enough to describe that the repeatability and stability of Ni/TiZrCrMnFeNi(800) were fairly good. In summary, the prepared high specific surface area of TiZrCrMnFeNi(800) can be used as an inactive/stable catalyst support in liquid-phase hydrogenation without the leaching of constituent elements. In addition, we demonstrated that the prepared TiZrCrMnFeNi(800) was more superior catalyst support than the conventional oxide supports of TiO_2_ and ZrO_2_. The superiority could be due to a lower work function of the CrMnFeNi alloy support than due to the loaded Ni species, the property that could work to promote the formation of active metallic Ni on the surface of Ni/TiZrCrMnFeNi(800).

**Table 4:** Comparison of rate constants (*k*) and normalized constants (*k*′) by the loading amounts of nickel among supported/unsupported Ni catalysts for 4-NP reduction

Sample	Temp. [°C]	Ni loading amount [wt%]	Reaction conditions	*k* [min^−1^]	*k*′ [min^−1^ mg_Ni_^−1^]	Ref.
Ni/TiZrCrMnFeNi(800)	25	10	4-NP (1.6 mM)	0.046	0.046	This study
Ni/TiO_2_		10	NaBH_4_ (47 mM)	0.015	0.015	
Ni/ZrO_2_		10	10 mg-cat/9 mL	0.003	0.003	
Ni/SNTs	Room temp.	23.0	4-NP (0.2 mM)	5.0	5.5	37
		15.2	NaBH_4_ (68 mM)	1.2	2.0	
		8.1	4 mg-cat/3 mL	0.59	1.8	
		5.6		0.46	2.0	
Ni/CNTs		25.3		0.10	0.1	
Ni/SNTs (wet impregnation)		26.2		0.16	0.2	
Ni/CB	30	0.2	4-NP (0.5 mM)	0.014	6.80	38
		11	NaBH_4_ (51 mM)	0.363	3.30	
		22	1 mg-cat/52 mL	0.597	2.71	
		33		0.392	1.19	
		41		0.065	0.16	
		49		0.038	0.08	
Unsupported Ni		n.d.		0.085	0.09	
RANEY® Ni	20	n.d.	4-NP (0.1 mM)	0.019	0.0066	39
Ni nanoparticles			NaBH_4_ (18 mM)	0.14	0.048	
			3 mg-cat/3 mL			

## Conclusions

A high specific surface area (23 m^2^ g^−1^) TiZrCrMnFeNi alloy nanopowder with a main hexagonal crystal structure was prepared at 800 °C using a molten salt synthesis method. Several analyses revealed the formation of CrMnFeNi alloy nanopowder passivated by the TiO_*x*_–ZrO_*y*_ surface shell, denoted as TiO_*x*_–ZrO_*y*_/CrMnFeNi. The prepared powder was stable and worked more effectively as a catalyst support in the hydrogenation of *p*-nitrophenol at 25 °C than common TiO_2_ and ZrO_2_. Thus, a potential application of TiZrCrMnFeNi alloy nanopowder as catalyst support was demonstrated.

## Author contributions

Yasukazu Kobayashi: conceptualization, supervision, funding acquisition, investigation, methodology, data curation, writing – original draft, writing – review, and editing. Shota Yokoyama: investigation, methodology, data curation, writing – review and editing. Ryo Shoji: methodology, writing – review and editing.

## Conflicts of interest

There are no conflicts to declare.

## Supplementary Material

RA-013-D3RA01797D-s001
