# Supplementary material for: Molten salt synthesis of CrMnFeNi alloy nanopowder passivated by TiOx–ZrOy shell used as a superior catalyst support in liquid-phase hydrogenation
Source: RSC Adv. 2023 Apr 5;13(16):10790–9. doi: 10.1039/d3ra01797d (PMC10074232; doi:10.1039/d3ra01797d)
Supplement: RA-013-D3RA01797D-s001 [file RA-013-D3RA01797D-s001.pdf]

ARTICLE

Electronic Supplementary Information

Molten Salt Synthesis of CrMnFeNi Alloy Nanopowder Passivated by TiO_x-ZrO_y Shell Used as a Superior Catalyst Support in Liquid-phase Hydrogenation

Yasukazu Kobayashi,*[a] Shota Yokoyama[b] and Ryo Shoji[b]

[a] *A Renewable Energy Research Centre, National Institute of Advanced Industrial Science and Technology, 2-2-9 Machiikedai, Koriyama, Fukushima 963-0298, Japan.*

Corresponding author, E-mail: yasu-kobayashi@aist.go.jp.

[b] *Department of Chemical Science and Engineering, National Institute of Technology, Tokyo College, 1220-2 Kunugida, Hachioji, Tokyo 193-0997, Japan.*

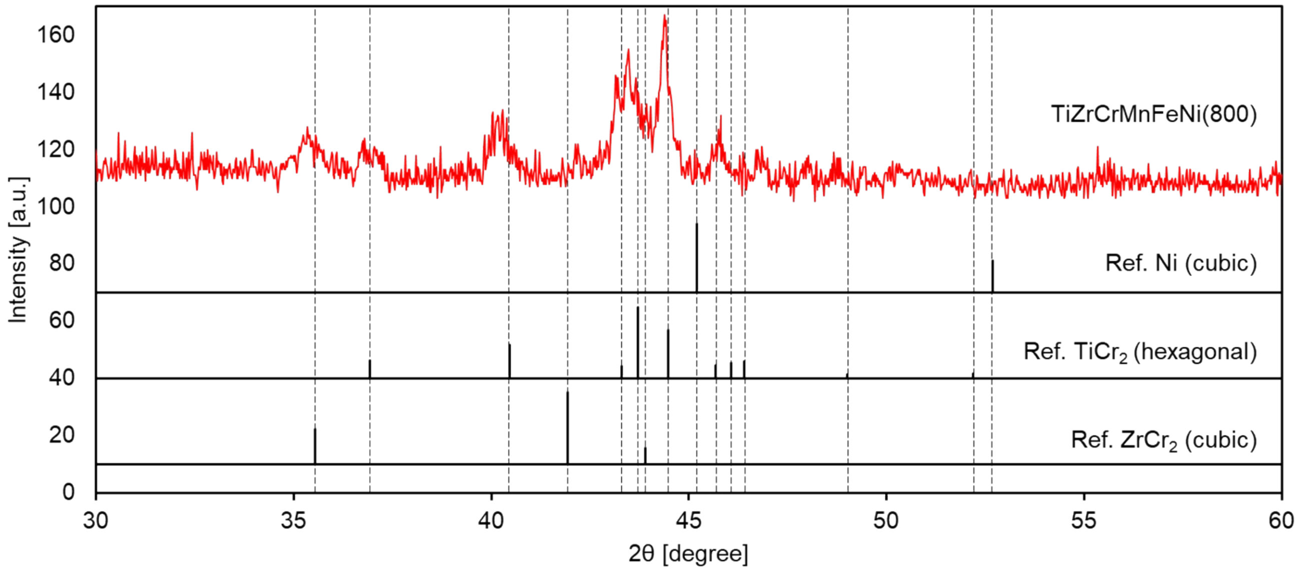

Fig. S1 XRD patterns for TiZrCrMnFeNi(800).

This journal is © The Royal Society of Chemistry 20xx

Table S1 Space group and crystal system for Laves phase intermetallic compounds.

Intermetallic compound	Space group	Crystal system
$TiCr_2$	P63/mmc	hexagonal
$TiMn_2$	P63/mmc	hexagonal
$TiFe_2$	P63/mmc	hexagonal
$TiNi_2$	n.d.	n.d.
$ZrCr_2$	Fd3(−)m	cubic
$ZrMn_2$	Fd3(−)m	cubic
$ZrFe_2$	P63/mmc	hexagonal
$ZrNi_2$	Fd3(−)m	cubic

This journal is © The Royal Society of Chemistry 20xx

Please do not adjust margins

Please do not adjust margins

Table S2 Elemental weight ratios measured using XPS, SEM–EDX, and TEM–EDX.

Sample	Elemental weight ratio [wt%]									
	Method	Position	Ti	Zr	Cr	Mn	Fe	Ni	O	Average
TiZrCrMnFeNi(Pre)	XPS	#1	5.1	19.5	11.2	11.5	8.4	8.2	36.1	Ti/Zr/Cr/Mn/Fe/Ni/O =6.8/18.7/10.1/12.2/9.7/7.6/35.0
		#2	5.3	18.6	8.4	11.9	12.7	8.6	34.4	
		#3	10.1	17.8	10.7	13.1	7.9	5.9	34.4	
TiZrCrMnFeNi(800)	XPS	#1	13.3	35.7	3.6	3.6	3.1	3.0	37.6	Ti/Zr/Cr/Mn/Fe/Ni/O =12.7/33.5/5.3/4.1/3.9/2.3/38.1
		#2	14.0	34.2	8.0	3.1	4.1	0	37	
		#3	10.9	30.5	4.4	5.6	4.6	3.9	40.2	
	SEM-EDX	Overall	8.3	47.9	7.5	7.2	7.9	7.8	13.3	Ti/Zr/Cr/Mn/Fe/Ni/O =8.2/48.4/8.0/8.0/8.7/9.2/9.5
		#1	6.9	71.7	3.2	3.0	3.4	3.3	8.6	
		#2	9.8	41.1	10.0	9.1	10.2	10.6	9.2	
		#3	7.8	32.8	11.5	12.6	13.5	14.9	6.8	
	TEM-EDX	#1	9.0	12.1	59.9	7.3	10.3	1.5	0.0	Ti/Zr/Cr/Mn/Fe/Ni/O =13.8/40.2/12.8/9.0/9.1/10.9/4.2
		#2	1.5	1.5	13.8	26.3	27.6	28.9	0.4	
		#3	53.6	16.9	24.1	1.7	3.1	0.6	0.0	
		#4	3.2	4.0	11.7	26.0	26.3	28.0	0.8	
		#5	28.3	33.7	25.8	3.2	5.8	1.6	1.7	
		#6	4.0	7.7	66.7	8.4	11.8	1.4	0.0	
		#7	1.9	5.8	7.0	29.5	19.8	31.7	4.3	
		#8	3.4	10.2	18.7	20.0	25.8	17.1	4.9	
		#9	3.7	10.0	8.5	24.8	20.0	26.0	7.1	
		#10	5.8	92.6	0.1	0.1	0.3	0.4	0.7	
		#11	17.6	27.6	3.6	15.1	15.4	17.0	3.7	
		#12	76.9	19.7	0.3	0.4	0.5	0.5	1.7	
		#13	5.6	88.2	0.2	0.2	0.4	0.2	5.3	
		#14	4.8	47.0	0.5	2.3	3.0	35.3	7.2	
		#15	10.5	31.3	8.9	13.7	14.4	16.6	4.5	
		#16	29.4	46.9	0.6	1.1	1.7	2.3	17.9	
		#17	2.4	42.4	44.2	6.7	3.3	1.0	0.0	
		#18	12.9	28.4	8.1	16.3	16.9	14.3	3.2	
		#19	16.6	76.7	0.2	0.2	0.4	0.1	5.7	
		#20	4.8	46.6	0.5	2.4	3.2	36.4	6.1	
		#21	8.8	33.2	22.9	13.7	12.4	9.0	0.0	
		#22	11.6	80.9	0.1	0.4	0.4	0	7	
		#23	6.5	85.8	0.2	0.1	0.3	0.3	6.9	
		#24	9.6	42.8	5.1	12.9	13.4	12.9	3.2	
		#25	13.8	76.7	0.2	0.3	0.5	0.1	8.4	
		#26	13.2	76.9	0.3	0.3	0.6	0.2	8.5	

This journal is © The Royal Society of Chemistry 20xx

Please do not adjust margins

Please do not adjust margins

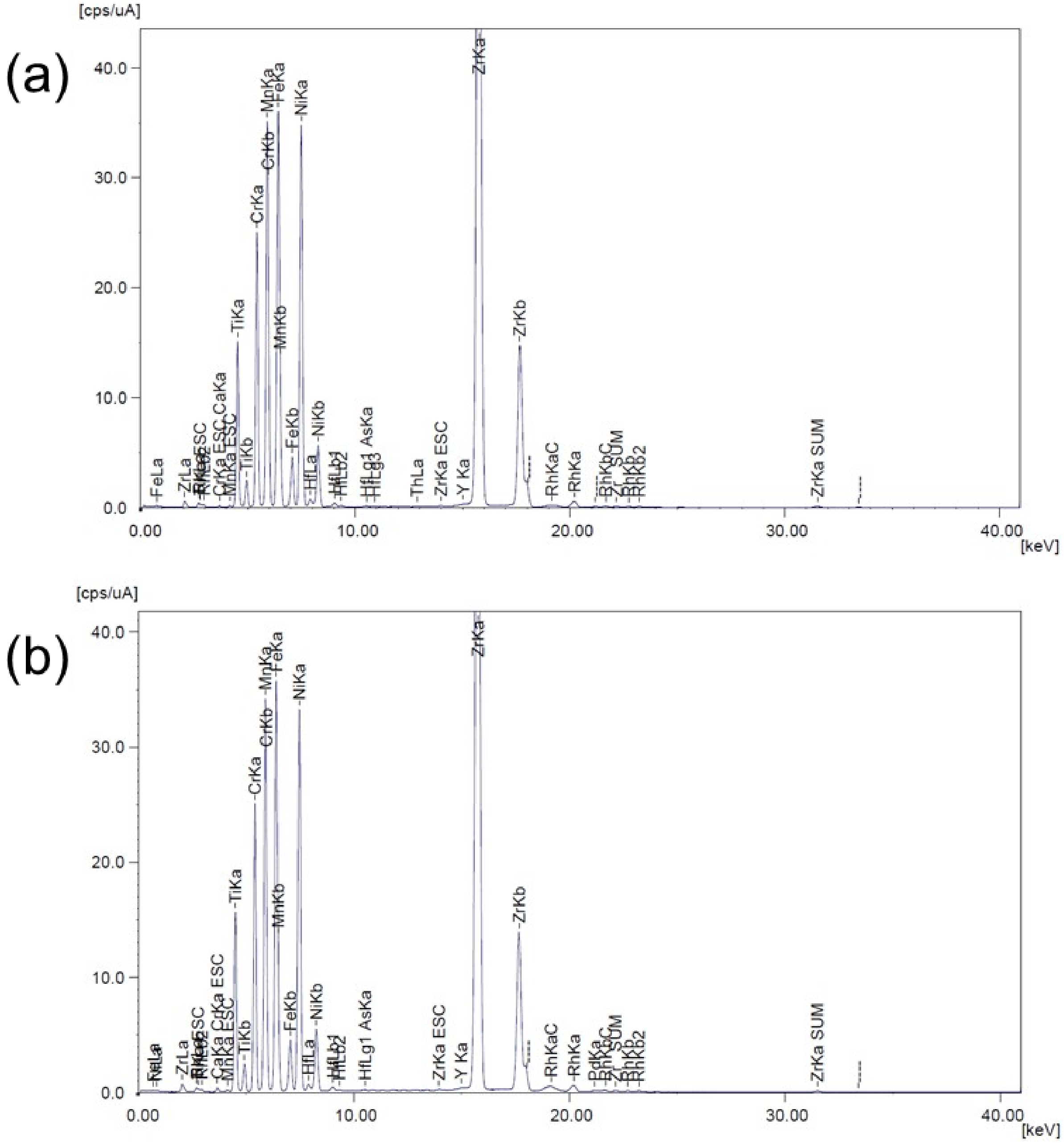

Fig. S2 EDX spectrum measured by EDX-8000 for TiZrCrMnFeNi(Pre) and TiZrCrMnFeNi(800). Molar ratios of Ti/Zr/Cr/Mn/Fe/Ni are summarized in Table 1.

Please do not adjust margins

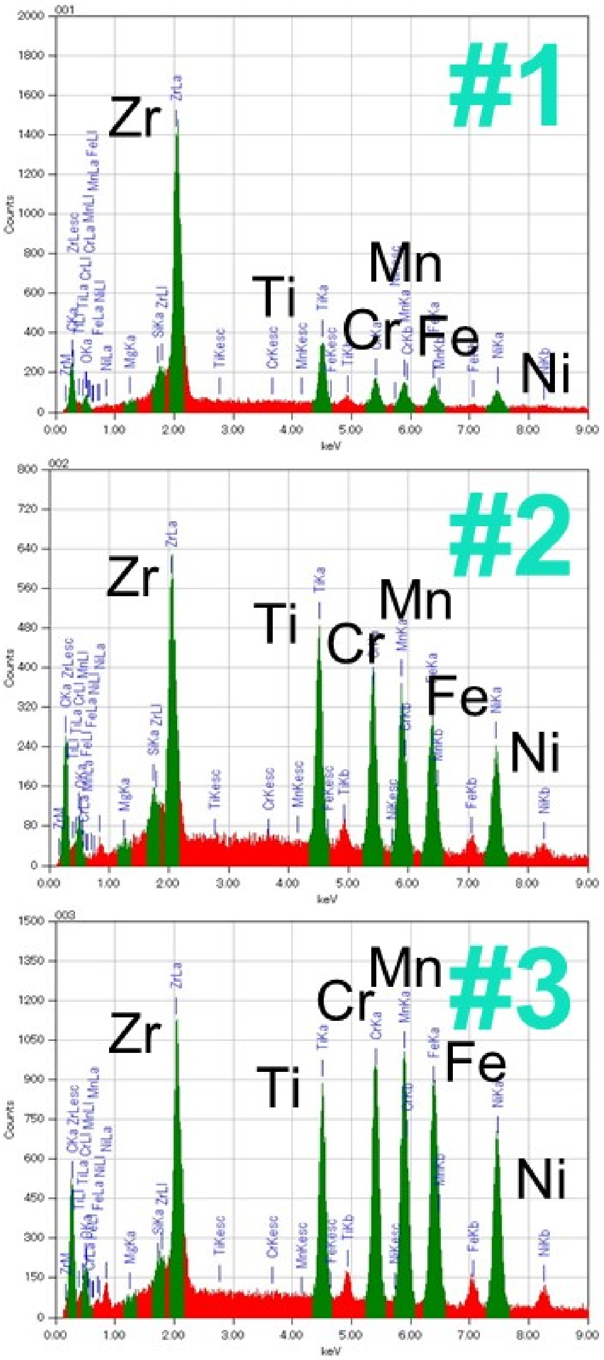

Fig. S3 EDX spectra for position #1–#3 corresponding to a SEM image of TiZrCrMnFeNi(800) in Fig. 2.

This journal is © The Royal Society of Chemistry 20xx

Please do not adjust margins

Please do not adjust margins

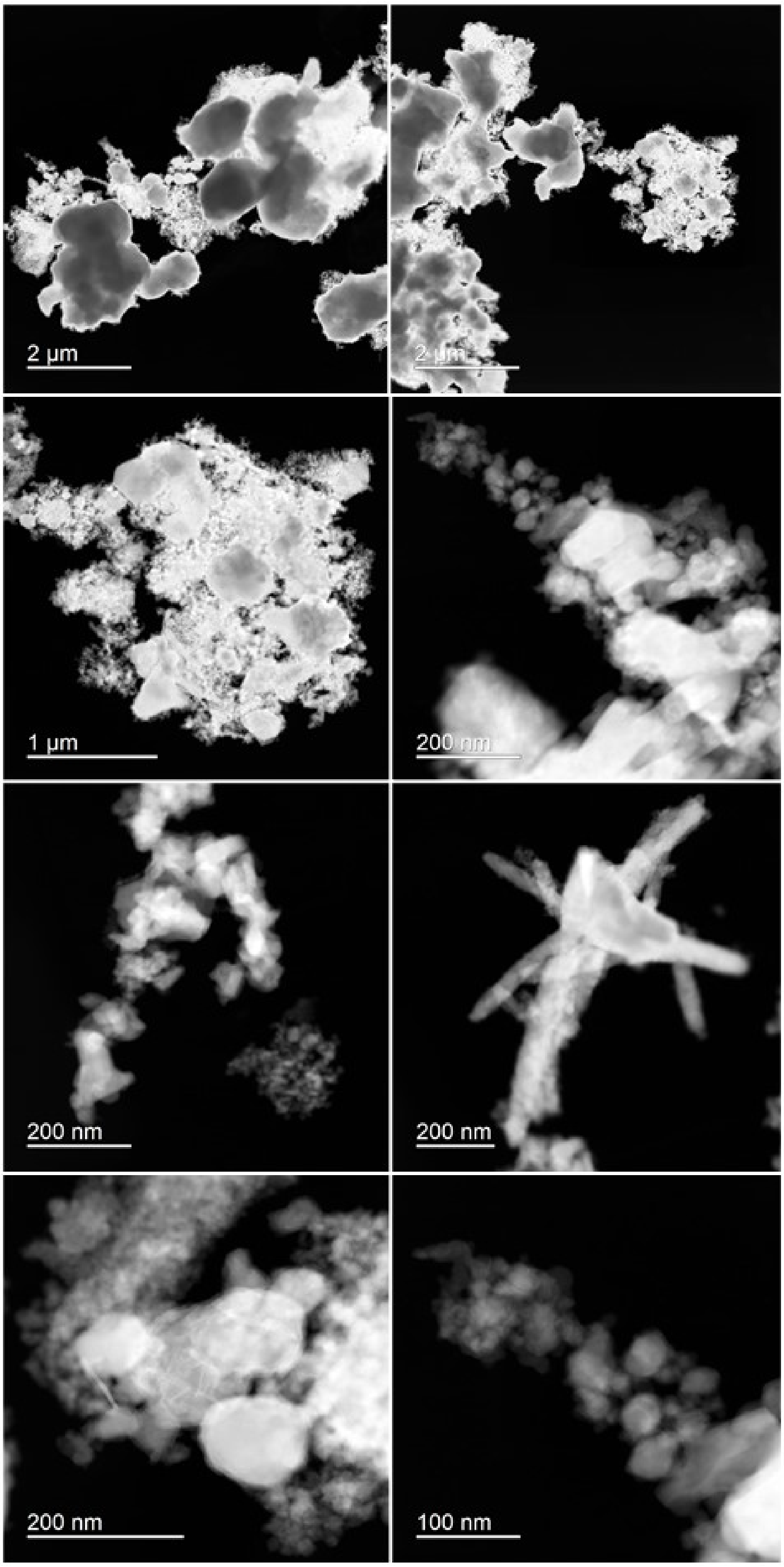

Fig. S4 TEM images for TiZrCrMnFeNi(800).

Please do not adjust margins

Table S3 Oxygen contents in TiZrCrMnFeNi(800) analyzed by LECO TCH-600.

Sample	No.	Used weight [mg]	Oxygen content [wt%]
TiZrCrMnFeNi(800)	1	10.7	4.38
	2	10.7	4.41
	3	10.1	4.42

This journal is © The Royal Society of Chemistry 20xx

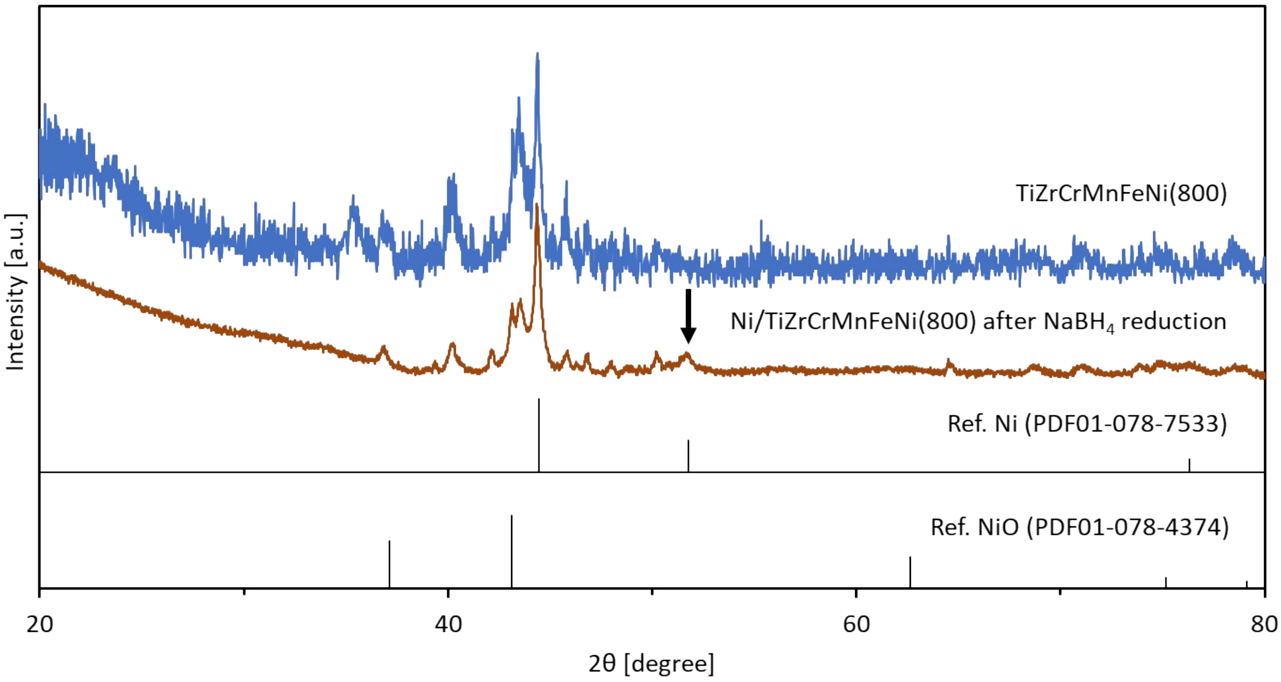

Fig. S5 XRD patterns for TiZrCrMnFeNi(800) and Ni/TiZrCrMnFeNi(800) after reduced by $NaBH_4$ at 25°C for 30 min with references of Ni and NiO. An arrow indicates a position of 51.8° corresponding to a signal for a (2 0 0) plane of metallic Ni.
